# Supplementary material for: Attitudes of medical students in Khartoum, Sudan towards the doctor-patient relationship: a cross-sectional study
Source: PeerJ. 2023 Jul 3;11:e15434. doi: 10.7717/peerj.15434 (PMC10324596; doi:10.7717/peerj.15434)
Supplement: Supplemental Information 1 [file peerj-11-15434-s001.pdf]

Attitudes of medical students in Khartoum, Sudan towards the doctor-patient relationship: a cross-sectional study

* Required

1. Age *

2. Gender *

Mark only one oval.

- Male(0)
- Female(1)

3. Study year *

Mark only one oval.

- 3rd year(Batch 93) (0)
- 4th year(Batch 92) (1)
- 5th year(Batch 91) (2)
- 6th year(Batch 94) (3)

The statements below refer to beliefs that people might have concerning doctors, patients and medical care. Read each item and then indicate how much you agree or disagree. Each of the statements is on a scale of 1 to 6.

Credits: the tool below is known as the PPOS and has been devised by Professor Edward Krupat of the Harvard Medical School. Copyrights ownership and authorship belong to him.

4. 1-The doctor is the one who should decide what gets talked about during a visit. *

Mark only one oval per row.

	6 Strongly disagree	5 Moderately disagree	4. Slightly disagree	3 Slightly agree	2 Moderately agree	1 Strongly agree
**Row 1**	◯	◯	◯	◯	◯	◯

5. 2-Although health care is less personal these days, this is a small price to pay for medical advances. *

Mark only one oval per row.

	6 Strongly disagree	5 Moderately disagree	4. Slightly disagree	3 Slightly agree	2 Moderately agree	1 Strongly agree
**Row 1**	◯	◯	◯	◯	◯	◯

6. 3-The most important part of the standard medical visit is the physical examination. *

Mark only one oval per row.

	6 Strongly disagree	5 Moderately disagree	4. Slightly disagree	3 Slightly agree	2 Moderately agree	1 Strongly agree
**Row 1**	◯	◯	◯	◯	◯	◯

7. 4-It is often best for patients if they do not have a full explanation of their medical condition. *

Mark only one oval per row.

	6 Strongly disagree	5 Moderately disagree	4. Slightly disagree	3 Slightly agree	2 Moderately agree	1 Strongly agree
**Row 1**	◯	◯	◯	◯	◯	◯

8. 5-Patients should rely on their doctors' knowledge and not try to find out about their conditions on their own. *

Mark only one oval per row.

	6 Strongly disagree	5 Moderately disagree	4. Slightly disagree	3 Slightly agree	2 Moderately agree	1 Strongly agree
**Row 1**	◯	◯	◯	◯	◯	◯

9. 6-When doctors ask a lot of questions about a patient's background, they are prying too much into personal matters. *

Mark only one oval per row.

	6 Strongly disagree	5 Moderately disagree	4. Slightly disagree	3 Slightly agree	2 Moderately agree	1 Strongly agree
**Row 1**	◯	◯	◯	◯	◯	◯

10. 7-If doctors are truly good at diagnosis and treatment, the way they relate to patients is not that important. *

Mark only one oval per row.

	6 Strongly disagree	5 Moderately disagree	4. Slightly disagree	3 Slightly agree	2 Moderately agree	1 Strongly agree
**Row 1**	◯	◯	◯	◯	◯	◯

11. 8-Many patients continue asking questions even though they are not learning anything new. *

Mark only one oval per row.

	6 Strongly disagree	5 Moderately disagree	4. Slightly disagree	3 Slightly agree	2 Moderately agree	1 Strongly agree
**Row 1**	◯	◯	◯	◯	◯	◯

12. 9-Patients should be treated as if they were partners with the doctor, equal in power and status. *

Mark only one oval per row.

	6 Strongly disagree	5 Moderately disagree	4. Slightly disagree	3 Slightly agree	2 Moderately agree	1 Strongly agree
**Row 1**	◯	◯	◯	◯	◯	◯

Untitled Section

13. 10-Patients generally want reassurance rather than information about their health. *

Mark only one oval per row.

	6 Strongly disagree	5 Moderately disagree	4. Slightly disagree	3 Slightly agree	2 Moderately agree	1 Strongly agree
**Row 1**	◯	◯	◯	◯	◯	◯

14. 11- If a doctor's primary tools are being open and warm, the doctor will not have a lot of success. *

Mark only one oval per row.

	6 Strongly disagree	5 Moderately disagree	4. Slightly disagree	3 Slightly agree	2 Moderately agree	1 Strongly agree
**Row 1**	◯	◯	◯	◯	◯	◯

15. 12- When patients disagree with their doctor, this is a sign that the doctor does not have the patient's respect and trust. *

Mark only one oval per row.

	6 Strongly disagree	5 Moderately disagree	4. Slightly disagree	3 Slightly agree	2 Moderately agree	1 Strongly agree
**Row 1**	◯	◯	◯	◯	◯	◯

16. 13-A treatment plan cannot succeed if it is in conflict with a patient's lifestyle or values. *

Mark only one oval per row.

	6 Strongly disagree	5 Moderately disagree	4. Slightly disagree	3 Slightly agree	2 Moderately agree	1 Strongly agree
**Row 1**	◯	◯	◯	◯	◯	◯

17. 14-Most patients want to get in and out of the doctor's office as quickly as possible. *

Mark only one oval per row.

	6 Strongly disagree	5 Moderately disagree	4. Slightly disagree	3 Slightly agree	2 Moderately agree	1 Strongly agree
**Row 1**	◯	◯	◯	◯	◯	◯

18. 15-The patient must always be aware that the doctor is in charge. *

Mark only one oval per row.

	6 Strongly disagree	5 Moderately disagree	4. Slightly disagree	3 Slightly agree	2 Moderately agree	1 Strongly agree
**Row 1**	⬭	⬭	⬭	⬭	⬭	⬭

19. 16-It is not that important to know a patient's culture and background in order to treat the person's illness. *

Mark only one oval per row.

	6 Strongly disagree	5 Moderately disagree	4. Slightly disagree	3 Slightly agree	2 Moderately agree	1 Strongly agree
**Row 1**	⬭	⬭	⬭	⬭	⬭	⬭

20. 17-Humor is a major ingredient in the doctor's treatment of the patient. *

Mark only one oval per row.

	6 Strongly disagree	5 Moderately disagree	4. Slightly disagree	3 Slightly agree	2 Moderately agree	1 Strongly agree
**Row 1**	⬭	⬭	⬭	⬭	⬭	⬭

21. 18-When patients look up medical information on their own, this usually confuses more than it helps. *

Mark only one oval per row.

	6 Strongly disagree	5 Moderately disagree	4. Slightly disagree	3 Slightly agree	2 Moderately agree	1 Strongly agree
**Row 1**	⬭	⬭	⬭	⬭	⬭	⬭
